# Young widowhood as a predictor of HIV risk behaviors in a high HIV prevalence setting in Siaya County, Kenya

**DOI:** 10.1080/16549716.2026.2684829

**Published:** 2026-06-17

**Authors:** Jackline A. Odhiambo, Louisa N. Ndunyu, Erick O. Nyambedha, Ushma D. Upadhyay, Craig R. Cohen, Sheri D. Weiser, Daniel Kwaro, Moses Otieno, Vivienne Kamire, Elizabeth A. Bukusi

**Affiliations:** a School of Public Health and Community Development, Maseno University, Maseno, Kenya; b Nyanam Widows Rising, Kisumu, Kenya; c Department of Sociology and Anthropology, Maseno University, Maseno, Kenya; d Department of Obstetrics, Gynecology & Reproductive Sciences, University of California San Francisco, San Francisco, CA, USA; e Center for Gender and Health Justice, University of California Global Health Institute, San Francisco, CA, USA; f Department of Obstetrics and Gynecology, School of Medicine, Maseno University, Maseno, Kenya; g Division of HIV, Infectious Disease and Global Medicine, University of California San Francisco, San Francisco, CA, USA; h Centre for Global Health Research, Kenya Medical Research Institute, Kisumu, Kenya; i Centre for Microbiology Research, Kenya Medical Research Institute, Kisumu, Kenya

**Keywords:** Widow inheritance, sexual cleansing, intergenerational sex, multiple sexual partners, transactional sex

## Abstract

**Background:**

Widowed women in sub-Saharan Africa bear a disproportionate burden of HIV/AIDS.

**Objectives:**

We examined the likely period of HIV exposure among widows living with HIV and the patterns and predictors of widowed women’s HIV risk behaviors.

**Methods:**

We performed a cross-sectional survey with an age-stratified random sample of widowed women in Siaya County, Kenya. We estimated the period of HIV exposure and the prevalence of HIV-risk behaviors, and used Firth’s penalized multivariable logistic regression to determine the association of socio-economic characteristics with HIV risk behaviors since widowhood.

**Results:**

Of the 480 widowed women, 178 (37.1%) had HIV. Of the 168 widows with confirmed dates of HIV diagnosis, 51.2% and 36.9% may have been exposed 2 and 5 years after widowhood, respectively. At least one in two widows engaged in condomless sex since widowhood. Widowhood during reproductive age was associated with higher odds of culturally sanctioned sexual behaviours. Specifically, young widowhood (<30 years old) was associated with condomless sex (AOR: 64.5, 95%CI: 15.0–277.1), widow inheritance (AOR: 34.5, 95%CI: 9.0–133.0), sexual cleansing (AOR: 41.5, 95%CI: 10.1–171.0), sex with a 10-year‑older partner (AOR: 227.4, 95%CI: 12.0–4324.5), and sex with an inheritor who had a wife (AOR: 16.8, 95%CI: 2.8–100.8). Living with HIV was associated with condomless sex (AOR: 2.9, 95%CI: 1.5–5.5), widow inheritance (AOR: 3.4, 95%CI: 1.8–6.5) and sexual cleansing (AOR: 3.2, 95%CI: 1.7–6.2).

**Conclusions:**

One in three widows living with HIV may have acquired the virus during widowhood. Young widowhood and living with HIV were associated with higher HIV risk behaviours. HIV testing, pre-exposure prophylaxis, and condom use in culturally sanctioned sexual practices should be studied further.

## MUHTASARI

**Usuli:**

Wanawake wajane katika Afrika Kusini mwa Jangwa la Sahara wana kiwango kikubwa cha maambukizo ya virusi vya ukimwi (VVU).

**Nia:**

Tulichunguza kipindi cha kuambukizwa VVU miongoni mwa wajane wanaoishi na VVU, na mifumo na vitabiri vya tabia za hatari zinazozidisha VVU kwa wanawake wajane.

**Mbinu:**

Tulifanya uchunguzi na sampuli ya wajane kutoka Kaunti ya Siaya Kenya waliochagulia kulingana na vikundi vya umri. Tulitumia takwimu za maelezo kukadiria kipindi cha maambukizo na VVU, na tabia hatarishi za kueneza VVU. Tulibainisha uhusiano kati ya sifa za kijamii na kiuchumi na tabia nyingi hatarishi za VVU tangu ujane.

**Matokeo:**

Kati ya wajane 480, 178 (37.1%) walikuwa na VVU. Miongoni mwa wajane 168 waliothibitisha tarehe za utambuzi wa VVU, 51.2% na 36.9% huenda waliambukizwa VVU katika ujane katika kipindi cha miaka miwili na tano baada ya ujane, kwa mtiririko huo. Angalau mmoja kati ya wajane wawili alihusiana na ngono bila kondomu tangu ujane. Ujane mchanga (miaka < 30 na 30–49) ulihusika na tabia nyingi hatarishi za VVU tangu ujane. Hasa ujane mchanga wa miaka < 30 ulihusishwa na ngono bila kondomu (AOR: 64.5, 95%CI: 15.0–277.1), urithi wa wajane (AOR: 34.5, 95%CI: 9.0–133.0), utakaso wa kijinsia (AOR: 41.5, 95%CI: 10.1–171.0), ngono na mpenzi mkubwa wa zaidi ya miaka kumi (AOR: 227.4, 95%CI: 12.0–4324.5), na ngono na mrithi aliyekuwa na mkewe mwenyewe (AOR: 16.8, 95%CI: 2.8–100.8). Wajane wanaoishi na VVU walihusishwa na ngono bila kondomu (AOR: 2.9, 95%CI: 1.5–5.5), urithi wajane (AOR: 3.4, 95%CI: 1.8–6.5) na utakaso wa kijinsia (AOR: 3.2, 95%CI: 1.7–6.2).

**Hitimisho:**

Angalau mmoja kati ya wajane watatu walio na VVU walipata VVU wakati wa ujane. Ujane mchanga na kuwa na VVU ulihusika na tabia nyingi hatarishi za VVU tangu ujane. Upimaji wa VVU, uzuiaji wa VVU na madawa, na matumizi ya kondomu katika utamaduni zinazohusisha ngono yanapaswa kukuzwa na kuchunguzwa zaidi.

## Paper Context

- **Main findings:** At least half of widows engaged in risky sexual behaviours since widowhood, with young widowhood and living with human immunodeficiency virus associated with multiple risky sexual behaviours.
- **Added knowledge:** Young widowhood is a critical period of human immunodeficiency virus risk among women, with at least one in three widows living with human immunodeficiency virus likely acquiring the virus during widowhood.
- **Global health impact for policy and action:** Human immunodeficiency virus prevention strategies among young widows with biomedical (preexposure prophylaxis, condom use) and social (alternative remarriage) interventions require testing.

## Background

Since the onset of the HIV pandemic in sub-Saharan Africa (SSA), widowhood has been significantly associated with HIV prevalence [1–7]. In recent population-based HIV impact assessments in several SSA countries, widowed women of reproductive age have had an HIV prevalence of 25–73%, higher than that of women who never married (3–23%), women who were married or living with a partner (4–28%), and women who are divorced or separated (12–56%) [8]. This disproportionate HIV burden among widowed women is also seen in Kenya, where the national HIV prevalence among widowed women of reproductive age is 35% and general adult widowed women is 26% [9]. In Kenya, the HIV prevalence among adult widowed women is higher than that of key populations such as men who have sex with men (17%), persons injecting drugs (20%) and similar to that of female sex workers (26%) [10]. While the high HIV prevalence among widowed women is well established, little is known about HIV risk behaviors during widowhood.

The majority of studies that have identified HIV risk behaviors in widowhood have been qualitative and have focused on cultural and economic factors [11–16]. Culturally sanctioned sexual practices such as sexual cleansing (the first sex after husband’s death believed to delink the widowed woman from the spirit of death) and widow inheritance (a temporary partnership of widowed women with male relatives in their husband’s clan) were associated with the spread of HIV within families in the early years of HIV pandemic, and painted widowed women as HIV carriers [12,14,17]. In the last decade of the 40-year HIV pandemic, widow inheritance has evolved to inheritance for sex by non-relatives or professional widow inheritors, a pattern associated with higher HIV prevalence than inheritance by relatives [11]. In addition, poverty due to loss of livelihoods, income or property makes widowed women vulnerable to transactional sex, sex work and forced sex, which increases their risk for HIV [13–15].

While a deceased husband’s HIV positivity creates an intractable link between HIV and widowhood [18,19], little is known about the proportion of widowed women with HIV who got infected after widowhood, and the predictors for engaging in HIV risk behaviors in this marginalized population. We provide a comprehensive quantitative assessment of HIV risk behaviors since widowhood, assessing the predictors of culturally sanctioned sexual practices, intergenerational sex, multiple sexual partnerships, and intimate partner violence among widowed women in Siaya County, Kenya. We also shed light on the potential period of HIV infection among widowed women with HIV.

## Methods

### Study setting, design and population

We conducted this study in Gem, one of the seven sub-counties in Siaya County in Kenya, where HIV prevalence stands at 15.3% [9]. We performed a cross-sectional survey to assess the predictors of HIV risk behaviors. The population of interest were widowed women in the Lake Victoria region in Kenya. A health and demographic surveillance system (HDSS) implemented by the Kenya Medical Research Institute (KEMRI) in Siaya County provided the study sampling frame [20–22]. We considered women who self-identified as widowed in the 2022 HDSS as eligible. We excluded widowed women who did not give informed consent, were unreachable after three attempts of recruitment, or were observed to have hearing problems, mental conditions, or other illnesses that prevented them from effective participation in the study.

### Sample size and sampling procedure

Based on the 2019 Kenyan census and an estimation of 17% of adult females in Gem sub-County to be widowed, the expected population of widows in Gem sub-County was 9400 [23,24]. Being a finite population, the sample size for the prevalence of HIV risk behaviours was calculated and adjusted using a finite population correction, and a 10% non-response rate. A 50% expected prevalence was used for a conservative sample size with a confidence level of 95% and margin of error of 5% using Cochran’s formula [25]. The calculated sample size was 410 widows.

$$n=\left(\left(Z2\times p\times \left(1-p\right)\right)/d2\right)=\left(\left(1.962\times 0.5\times \left(1-0.5\right)\right)/0.052\right)=384$$

$$n_{\mathrm{adjusted}}=n/\left(1+\left(\left(n-1\right)/N\right)\right)=384/\left(1+\left(383/9400\right)\right)=369$$

With 10% non-response n_final_ = n_adjusted_/(1–0.1) = 369/0.9 = 410

In addition to estimating the sample size for the prevalence of HIV risk behaviours, we also determined the sample size for logistic regression with a multiple predictors [26]. We used an events-per-variable criterion, assuming 15 events per predictor. With 15 predictors and an assumed outcome prevalence of 50%, we calculated a sample size that yielded approximately 450 participants. We increased this to 495 after adjusting for a 10% non-response rate.

$$N=\frac{\mathrm{EPV}\times p}{\pi }$$

Given the two sample sizes (410 widows for prevalence and 495 widows for association study), we proceeded with the largest sample size of 495. We took a computer-generated random sample of women who self-identified as widowed in the 2022 HDSS, stratified by age (29–39 years, 40–59 years, 60+ years). These age categories reflected meaningful differences in HIV risk profiles. Women aged 29–39 years were more likely to be recently widowed, sexually active, and in relationships influenced by economic vulnerability. Widows aged 40–59 years represented a mid-life cohort in which patterns of sexual activity and partnership formation may differ, while women aged 60 years and above were an older group assumed to have lower levels of sexual activity, hence risk behaviors.

### Data collection

We developed study questionnaires in English, Kiswahili and Dholuo, the local language, to measure socio-demographic and economic characteristics and HIV risk behaviors. We programmed Survey Solutions, a free software developed by the World Bank Group for data capture, with skip logic and validation rules, and installed it on tablets for front-end data collection to minimize data entry errors, reduce costs of data collection, and enable real‑time data monitoring [27]. Data collection occurred from July to August 2024.

We hired and trained a field coordinator and 10 female research assistants with prior experience conducting household surveys in Gem sub-County. We pretested and improved the survey tools with a sample of 28 widows from Alego and Bondo sub-Counties in Siaya. The HDSS team provided a confidential file with information such as names of individuals, their compounds and villages to facilitate locating the sampled participants. Village Reporters, who are community resource persons, helped locate the sampled participants for recruitment. Due to low language and technological literacy, and sensitivity of study questions, the research assistants administered face-to-face surveys with consenting participants. We also held weekly meetings with the field team to discuss field progress and challenges, and review Survey Solution reports for data quality and feedback.

### Measures

We measured 14 HIV risk behaviors since widowhood as binary outcomes. These outcomes were selected a priori and included culturally sanctioned sexual practices of widow inheritance, sexual cleansing and condomless sex. It also included intergenerational sex with a partner who was 10 or more years younger or older. The 10-year gap has mostly been used in studies with adolescent girls and young women to reflect generational or life‑stage differences, power and health‑risk differences, and enable comparison across studies [28]. Multiple sexual partnerships included an inheritor having two or more concurrent widows, widows having two or more sequential inheritors, and an inheritor having both a wife and a widow. Transactional sex included widows giving gifts for sex, receiving gifts for sex, or engaging in sex work. The final outcome was sex under the influence of drugs. We identified condomless sex as the primary outcome, and the remaining 13 outcomes as secondary outcomes, as they would all require condomless sex to increase the risk of HIV transmission.

The covariates, also selected a priori based on theoretical relevance and literature, included dates of birth, marriage and widowhood [24,29–32]. We calculated the age of the widowed woman in years and reported it in decades, the duration of her marriage in years (the difference between her year of marriage and year of widowhood), which we also reported in decades, and the duration of widowhood in years (the difference between the current year of data collection and her year of widowhood). We defined recent widowhood as being widowed within the last 2 years and grouped the rest of the duration of widowhood in decades. We also collected data on the women’s highest educational level, completion of a vocational school, household headship and size, whether she was in a polygamous marriage with the deceased husband, religion, current economic activity, and home and land ownership by self, deceased husband or marital relatives. Home ownership by widowed women meant that the woman had her home in her own compound. Marital relatives included the widows’ children, in-laws, or extended relations in her marital community.

We collected additional data on self-reported HIV status of widowed women and their deceased husbands, and estimated whether widowed women with HIV were infected before or after widowhood. We confirmed the widows’ HIV status and their date of HIV diagnosis with their medical records. Because the exact date of HIV acquisition is unobserved, we defined the likely HIV exposure period as occurring 2–5 years prior to the date of HIV diagnosis, consistent with surveillance and cohort studies estimating median delays of 2–5 years between infection and diagnosis of HIV in sub-Saharan Africa [33]. We used data on the date of HIV diagnosis, date of widowhood, and date of data collection to calculate the likely period of HIV exposure.

### Data analysis

We used Stata 17.0 (StataCorp, College Station, TX) for analysis and performed descriptive statistics, reporting numbers and percentages for socio-demographic and economic characteristics, and HIV risk behaviors. We conducted a bivariate analysis comparing the socio-demographic and economic characteristics with their HIV status and reported *p*-values.

To determine associations between socio-demographic and economic characteristics and the HIV risk behaviors, we first checked assumptions for the logistic regression models, including multicollinearity, events per variable, and model fit. Age, duration of marriage, and duration of widowhood—time-based covariates related to the life course—had moderate to high multicollinearity (variance inflation factors of 6–11). We thus calculated age at marriage (the difference between age and duration of marriage) and categorized it to reflect cultural conventions of early to late marriage (≤24 years, 25–29 years, and 30+ years). We also calculated age at widowed (the difference between age and duration of widowhood) and categorized it to reflect young to older widowhood (<30 years, 30–49 years, 50–59 years, 60+ years).

For missingness, a minimal 2–4% of participants were excluded from regression analyses for 12 out of 14 outcomes due to incomplete information. The remaining two outcomes had 7% outcome missingness, which drove regression missingness to 9%. We considered the missingness low to moderate and conducted complete-case analyses, reporting reduced N per variable across all results. Given small cell counts and quasi‑complete separation that inflated odds ratios for some variables when using standard logistic regression, we conducted bivariable and multivariable penalized logistic regressions with Firth’s correction to reduce bias and provide finite odds ratios. Because of the exploratory nature of the study, we considered all the covariates in multivariable regressions regardless of their statistical significance in the bivariable regression. This approach also minimized residual confounding. For the primary outcome (condomless sex), raw *p*-values (p) were reported with interpretations focused on effect sizes and 95%CIs. For the secondary outcomes, we accounted for multiple testing by adjusting *p*-values using the Benjamini–Hochberg false discovery rate (FDR) procedure. We report odds ratio (OR), adjusted odds ratio (AOR), 95% confidence interval (95%CI), with raw *p*-values (p) and FDR-adjusted *p*-values (q) considered significant at q < 0.05.

## Results

**Figure 1.**
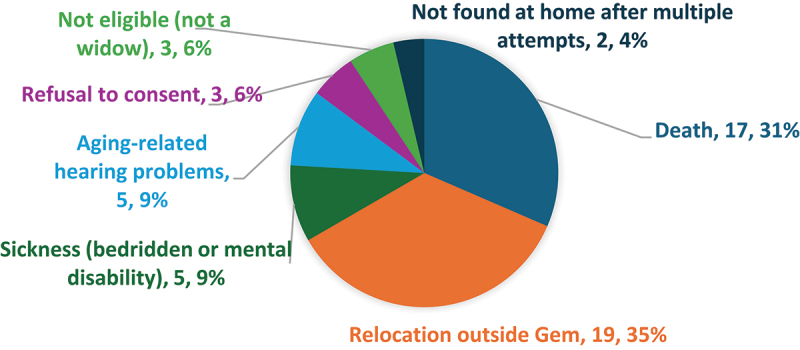
Reasons for non-consent or ineligibility of participants for this study (*N* = 56). A total of 536 participants were randomly sampled, of whom 480 (89.6%) participated and 56 (10.4%) did not participate.

Of the 536 randomly sampled participants, 480 (89.6%) were eligible for the study and provided informed consent. The remaining 56 (10.4%) did not participate in the study for various reasons, including death in the period between the HDSS survey in 2022 and the time of data collection in 2024 (17, 31.5%), and relocation (19, 35.2%) (Figure 1). The actual analytic sample (480) was below the adjusted target (495) but exceeded the minimum required sample size (450), suggesting the study retained adequate statistical power for the planned analyses.

The median age of the participants was 58 years (interquartile range (IQR): 50, 67) (Table 1). Most of the widowed women had completed primary school education (69.8%), had a household size of up to four persons (68.1%), and all were Christians involved in different religious denominations, including African traditional churches (28.0%), Catholic (15.7%), non-denominational (30.0 %), and protestants (26.1%). Only 16.0% and 11.0% owned their homes and agricultural land, respectively, with most living in homes (63.0%) and cultivating lands (54.0%) owned by their deceased spouse.

**Table 1.** Socio-demographic characteristics and their association with HIV status of widowed women in Gem, Siaya, Kenya.

	All widowed women	Widowed women without HIV	Widowed women with HIV	
	N = 480	N = 302	N = 178	
Variable	n (%)	n (%)	n (%)	p-value
Median age (IQR), years	58 (50, 67)	61 (53, 73)	53 (47, 59)	<0.001
Age categories, years				
29–39	23 (4.8)	11 (3.6)	12 (6.7)	<0.001
40–49	94 (19.6)	42 (13.9)	52 (29.2)	
50–59	157 (32.7)	80 (26.5)	77 (43.3)	
60–75	145 (30.2)	108 (35.8)	37 (20.8)	
>75	61 (12.7)	61 (20.2)	0 (0.0)	
Median duration of marriage (IQR), years	23 (13, 33)	27 (16, 38)	16 (9, 26)	<0.001
Duration of marriage categories, years	*N* = 472	*N* = 296	*N* = 176	
≤10	84 (17.8)	34 (11.5)	50 (28.4)	<0.001
11–20	125 (26.5)	63 (21.3)	62 (35.2)	
21–30	111 (23.5)	77 (26.0)	34 (19.3)	
>30	152 (32.2)	122 (41.2)	30 (17.0)	
Median duration of widowhood (IQR), years	16 (8, 23)	14 (7, 23)	17 (11, 22)	0.116
Duration of widowhood categories, years	*N* = 472	*N* = 296	*N* = 176	
≤2	15 (3.2)	10 (3.4)	5 (2.8)	<0.001
03–10	145 (30.5)	108 (36.2)	37 (20.9)	
11–20	176 (37.1)	90 (30.2)	86 (48.6)	
>20	139 (29.3)	90 (30.2)	49 (27.7)	
Age at marriage, years	*N* = 472	*N* = 296	*N* = 176	
≤24	76 (16.1)	50 (16.9)	26 (14.8)	0.148
25–29	94 (19.9)	66 (22.3)	28 (15.9)	
≥30	302 (64.0)	180 (60.8)	122 (69.3)	
Age at widowhood, years	*N* = 475	*N* = 298	*N* = 177	
<30	96 (20.2)	38 (12.8)	58 (32.8)	<0.001
30–49	251 (52.8)	149 (50.0)	102 (57.6)	
50–59	71 (15.0)	58 (19.5)	13 (7.3)	
≥60	57 (12.0)	53 (17.8)	4 (2.3)	
Highest level of education attained				
None	92 (19.2)	75 (24.8)	17 (9.6)	<0.001
Primary	335 (69.8)	197 (65.2)	138 (77.5)	
Secondary and above	53 (11.0)	30 (9.9)	23 (12.9)	
Completed other type of vocational school	56 (11.7)	31 (10.3)	25 (14.0)	0.213
Head of household	462 (96.3)	290 (96.0)	172 (96.6)	0.737
Median people living in household (IQR)	3 (2, 5)	3 (1, 5)	4 (2, 5)	0.002
People living in household categories				
4 and below	327 (68.1)	217 (71.9)	110 (61.8)	0.022
Above 4	153 (31.9)	85 (28.1)	68 (38.2)	
Polygamous marriage with deceased husband	248 (51.7)	158 (52.3)	90 (50.6)	0.71
Christian denominations			*N* = 177	
African Traditional Churches	134 (28.0)	45 (14.9)	30 (16.9)	0.312
Catholic	75 (15.7)	79 (26.2)	55 (31.1)	
Non-denominational churches	145 (30.3)	91 (30.1)	54 (30.5)	
Protestant	125 (26.1)	87 (28.8)	38 (21.5)	
Current economic activity				
Farming	241 (50.2)	149 (49.3)	92 (51.7)	0.004
Casual labor	43 (9.0)	26 (8.6)	17 (9.6)	
Employed	5 (1.0)	2 (0.7)	3 (1.7)	
Microbusiness	139 (29.0)	80 (26.5)	59 (33.1)	
Supported by others	52 (10.8)	45 (14.9)	7 (3.9)	
Home ownership				
Yourself	75 (16.0)	37 (12.3)	38 (21.3)	<0.001
Deceased spouse	304 (63.0)	214 (70.9)	90 (50.6)	
Marital relatives†	101 (21.0)	51 (16.9)	50 (28.1)	
Agricultural land ownership				
Yourself	51 (11.0)	29 (9.6)	22 (12.4)	0.007
Deceased spouse	258 (54.0)	179 (59.3)	79 (44.4)	
Marital relatives†	171 (35.6)	94 (31.1)	77 (43.3)	

IQR: Interquartile range; HIV: Human immunodeficiency virus; †Marital relatives: children, other family members such as in-laws or extended relations in the marital community.

### Likely period of HIV infection among widows living with HIV

Of the 480 widowed women, 178 (37.1%) had HIV and 105 (21.9%) had HIV‑positive husbands (Table 2). Among the 178 widowed women with HIV, 83 (46.6%) reported that their husbands were living with HIV. Of the 168 widows who provided the date of their HIV diagnosis from medical records, 67.9% were diagnosed with HIV after being widowed. Assuming HIV exposure occurred 2 years before diagnosis, then 86 (51.2%) of the widows living with HIV may have acquired HIV after widowhood. Assuming HIV exposure occurred 5 years before diagnosis, then 36.9% (*n* = 62) of widowed women living with HIV may have acquired HIV after widowhood.

**Table 2.** HIV status and likely period of HIV acquisition among widowed women living with HIV in Gem, Siaya, Kenya.

Variable	n (%)
Widow’s HIV status (*N* = 478)	
Negative	301 (62.9)
Positive	178 (37.1)
Deceased husband’s HIV status (*N* = 480) ‡	
Negative	288 (60.0)
Positive	105 (21.9)
Don’t know	87 (18.1)
Deceased husband’s HIV status among HIV positive widows (*N* = 178)	
Negative	47 (26.4)
Positive	83 (46.6)
Don’t know	48 (27.0)
Calculated period of HIV diagnosis (*N* = 168) †	
Diagnosed before widowhood	54 (32.1)
Diagnosed after widowhood	114 (67.9)
Self-reported period of HIV diagnosis (*N* = 171)	
Before or within six months of husband’s death	71 (41.5)
More than 6 months after husband death	100 (58.5)
Estimated period of HIV infection (*N* = 168)	
Assuming HIV exposure occurred 2 years before diagnosis	
Exposed before widowhood	82 (48.8)
Exposed after widowhood	86 (51.2)
Assuming HIV exposure occurred 5 years before diagnosis	
Exposed before widowhood	106 (63.1)
Exposed after widowhood	62 (36.9)

HIV: Human immunodeficiency virus.

^‡^Self-reported by the widows.

^†^Calculated by subtracting the date of HIV diagnosis from the date of widowhood. If negative value, then HIV diagnosis occurred before widowhood. If positive, then HIV diagnosis occurred after widowhood.

### HIV risk and behaviors since widowhood

**Figure 2.**
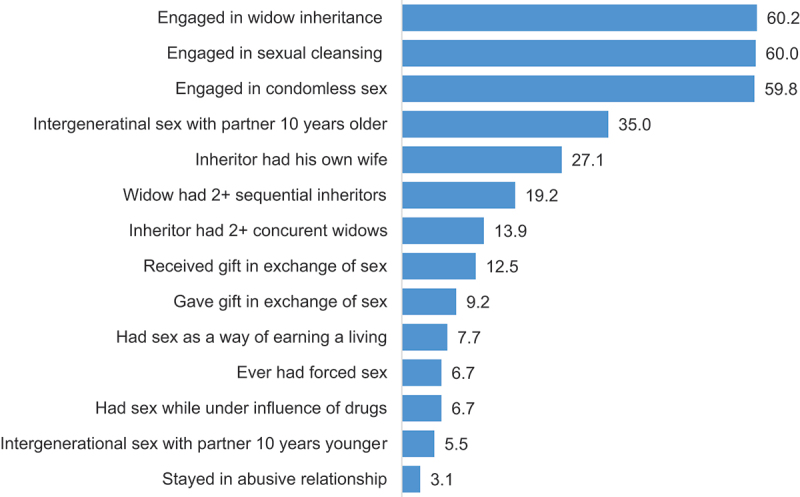
Period prevalence of HIV risk behaviors since widowhood among widowed women in Gem, Siaya, Kenya, ordered from highest to lowest prevalence (*N* = 480).

The most common HIV risk behaviors among the widowed women were culturally sanctioned sexual practices of widow inheritance (60.2%) and sexual cleansing (60.0%), both of which were strongly correlated with condomless sex (55.6%) (Figure 2). This was followed by intergenerational sex with partners who were 10 years older (35.0%) and multiple sexual partnerships where the widowed women had two or more sequential inheritors (19.2%). The least reported HIV risk behavior was intimate partner violence, defined as being in an abusive relationship (3.1%).

### Risk factors for HIV risk behaviors

Bivariable results for factors associated with HIV risk behaviors can be found in Supplementary Tables 1a, 1b, and  1c. Age at marriage, age at widowhood, education level, household size, Christian denomination, current economic activity, and home ownership were associated with culturally sanctioned sexual practices (widow inheritance, sexual cleansing, condomless sex) (Supplementary Table 1a). For intergenerational sex, age at marriage, age at widowhood, and household size were associated with sex with 10-year older partners while current economic activity and home ownership were associated with sex with 10-year younger partners.

For multiple sexual partnerships, age at marriage and age at widowhood were associated with an inheritor having two or more concurrent widows (Supplementary Table 1b). Age at marriage, age at widowhood, education level, current economic activity, and the deceased husband’s HIV status were associated with a widow having two or more sequential inheritors. Age at widowhood was also associated with a widow being with an inheritor who had his own wife. For intimate partner violence, only household headship was associated with staying in an abusive relationship. No variable was associated with forced sex.

For transactional sex, current economic activity and deceased husband’s HIV status were associated with widows receiving gifts for sex (Supplementary Table 1c). Age at widowhood and education level were also associated with widows receiving gifts for sex. Age at widowhood was also associated with sex work. No variable was associated with sex under the influence of drugs.

In the multivariable analysis, age at widowhood reached statistical significance in its association with four of the 14 HIV risk behaviors. For the primary outcome, women widowed at an age of <30 years (AOR: 64.5, 95%CI: 15.0–277.1, *p* < 0.001) and 30–49 years (AOR: 25.0, 95%CI: 7.2–86.1, *p* < 0.001) had higher odds of engaging in condomless sex than those widowed when above 60 years (Table 3). Compared to HIV‑negative widows, widowed women living with HIV also had higher odds of engaging in condomless sex (AOR: 2.9, 95%CI: 1.5–5.5, *p* < 0.001). Late marriage (marriage after 30 years of age) was also associated with higher odds of condomless sex than early marriage (marriage before 24 years of age) (AOR: 2.27, 95%CI: 1.10–4.70, *p* = 0.03). Completion of vocational school as associated with lower odds of condomless sex (AOR: 0.42, 95%CI: 0.20–0.88, *p* = 0.02). Finally, worshipping in traditional African churches was associated with condomless sex compared to Catholic churches (AOR: 2.47, 95%CI:1.10–5.51, *p* = 0.03).

**Table 3.** Multivariable association between socio-demographic characteristics and HIV risk behaviors among widows recruited from a high-HIV prevalence setting in Kenya.

	Culturally sanctioned sexual practices			Intergenerational sex	
	Widow inheritance *N* = 471	Sexual cleansing *N* = 471	Primary Outcome Condomless sex *N* = 471	10+ year younger sex partner *N* = 467	10+ year older sex partner *N* = 460
Variables	AOR (95% CI) p, q	AOR (95% CI) p, q	AOR (95% CI) p	AOR (95% CI) p, q	AOR (95% CI) p, q
Age at marriage, years					
≤24	Ref	Ref	Ref	Ref	Ref
25–30	1.04 (0.46–2.33) 0.92, 0.97	0.98 (0.44–2.20) 0.97, 0.99	1.19 (0.53–2.65) 0.68	1.45 (0.16–13.50) 0.74, 0.91	1.29 (0.56–2.95) 0.55, 0.83
>30	2.39 (1.14–5.00) 0.02, 0.20	2.32 (1.11–4.85) 0.02, 0.22	2.27 (1.10–4.70) 0.03	4.46 (0.62–31.95) 0.14, 0.50	1.21 (0.58–2.52) 0.61, 0.85
Age at widowhood, years					
<30	34.51 (8.95–133.03) <0.01, <0.01	41.50 (10.07–170.99) <0.01, <0.01	64.45 (14.99–277.06) <0.01	1.98 (0.09–42.24) 0.66, 0.87	227.42 (11.96–4324.47) <0.01, 0.01
30–49	19.02 (6.09–59.39) <0.01, <0.01	25.35 (7.41–86.72) <0.01, <0.01	25.03 (7.28–86.09) <0.01	1.68 (0.09–32.82) 0.73, 0.91	106.87 (5.96–1917.17) <0.01, 0.04
50–59	3.31 (1.03–10.64) 0.04, 0.27	4.43 (1.26–15.54) 0.02, 0.21	4.60 (1.31–16.16) 0.02	0.48 (0.01–26.41) 0.72, 0.91	25.36 (1.38–465.25) 0.03, 0.22
≥60	Ref	Ref	Ref	Ref	Ref
Highest level of education attained					
None	Ref	Ref	Ref	Ref	Ref
Primary	1.51 (0.74–3.10) 0.26, 0.62	1.43 (0.69–2.95) 0.33, 0.66	1.50 (0.73–3.07) 0.27	4.09 (0.23–71.27) 0.33, 0.66	0.70 (0.35–1.39) 0.31, 0.65
Secondary & above	0.52 (0.19–1.41) 0.20, 0.57	0.51 (0.19–1.37) 0.18, 0.55	0.56 (0.21–1.53) 0.26	3.66 (0.17–77.21) 0.40, 0.72	0.37 (0.14–0.94) 0.04, 0.25
Completed vocational school					
No	Ref	Ref	Ref	Ref	Ref
Yes	0.36 (0.17–0.76) 0.01, 0.14	0.37 (0.18–0.79) 0.01, 0.15	0.42 (0.20–0.88) 0.02	1.22 (0.40–3.69) 0.72, 0.91	0.48 (0.23–0.98) 0.04, 0.28
Head of household					
No	Ref	Ref	Ref	Ref	Ref
Yes	2.12 (0.47–9.56) 0.33, 0.66	2.10 (0.47–9.42) 0.33, 0.66	1.22 (0.27–5.52) 0.80	0.65 (0.02–20.66) 0.81, 0.93	5.19 (1.05–25.52) 0.04, 0.28
Household size					
4 and below	Ref	Ref	Ref	Ref	Ref
Above 4	1.35 (0.78–2.31) 0.28, 0.63	1.49 (0.87–2.57) 0.15, 0.51	1.37 (0.80–2.36) 0.25	1.01 (0.43–2.38) 0.98, 0.99	1.78 (1.12–2.81) 0.01, 0.18
Polygamous marriage with deceased husband					
No	Ref	Ref	Ref	Ref	Ref
Yes	0.80 (0.48–1.35) 0.41, 0.72	0.86 (0.52–1.45) 0.58, 0.85	0.89 (0.53–1.49) 0.65	0.92 (0.37–2.27) 0.86, 0.96	1.09 (0.69–1.71) 0.72, 0.91
Christian denominations					
Catholic	Ref	Ref	Ref	Ref	Ref
African traditional churches	2.84 (1.27–6.35) 0.01, 0.16	2.49 (1.12–5.54) 0.03, 0.22	2.47 (1.10–5.51) 0.03	0.55 (0.15–2.05) 0.37, 0.69	1.07 (0.53–2.18) 0.84, 0.95
Non-denominational churches	1.49 (0.71–3.16) 0.29, 0.64	1.47 (0.69–3.10) 0.31, 0.65	1.36 (0.64–2.88) 0.43	0.58 (0.18–1.86) 0.36, 0.69	0.73 (0.36–1.48) 0.39, 0.71
Protestant	0.89 (0.43–1.88) 0.77, 0.92	0.94 (0.45–1.98) 0.87, 0.96	0.85 (0.40–1.78) 0.66	0.60 (0.18–1.99) 0.41, 0.72	1.04 (0.51–2.11) 0.92, 0.97
Current economic activity					
Farming	Ref	Ref	Ref	Ref	Ref
Casual labor	0.80 (0.35–1.85) 0.60, 0.85	0.78 (0.34–1.79) 0.56, 0.84	0.63 (0.27–1.46) 0.28	4.06 (1.35–12.20) 0.01, 0.17	1.23 (0.60–2.53) 0.57, 0.84
Employed	1.77 (0.22–14.18) 0.59, 0.85	1.69 (0.21–13.89) 0.63, 0.86	1.57 (0.21–11.71) 0.66	4.69 (0.54–40.87) 0.16, 0.53	0.68 (0.09–5.29) 0.71, 0.90
Microbusiness	1.05 (0.59–1.86) 0.88, 0.95	0.90 (0.51–1.60) 0.72, 0.91	1.08 (0.61–1.92) 0.80	0.97 (0.35–2.63) 0.94, 0.97	0.95 (0.58–1.56) 0.85, 0.95
Supported by others††	1.38 (0.53–3.62) 0.51, 0.80	1.31 (0.49–3.50) 0.59, 0.85	1.25 (0.46–3.37) 0.66	0.68 (0.04–11.92) 0.79, 0.93	3.05 (1.08–8.63) 0.04, 0.25
Home ownership					
Yourself	Ref	Ref	Ref	Ref	Ref
Deceased spouse	0.37 (0.14–0.95) 0.04, 0.27	0.40 (0.16–1.02) 0.05, 0.30	0.46 (0.18–1.16) 0.10	0.40 (0.13–1.19) 0.10, 0.42	1.29 (0.64–2.59) 0.48, 0.78
Marital relatives†	0.45 (0.15–1.32) 0.14, 0.51	0.49 (0.17–1.42) 0.19, 0.57	0.60 (0.21–1.73) 0.34	0.47 (0.13–1.71) 0.25, 0.61	0.96 (0.44–2.10) 0.92, 0.97
Agricultural land ownership					
Yourself	Ref	Ref	Ref	Ref	Ref
Deceased spouse	0.58 (0.21–1.63) 0.30, 0.64	0.62 (0.23–1.72) 0.36, 0.69	0.48 (0.17–1.35) 0.16	0.87 (0.24–3.10) 0.83, 0.94	0.60 (0.26–1.38) 0.23, 0.59
Marital relatives†	1.69 (0.21–13.89) 0.63, 0.86	1.57 (0.21–11.71) 0.66, 0.88	4.69 (0.54–40.87) 0.16	0.68 (0.09–5.29) 0.71, 0.90	1.51 (0.06–35.24) 0.80, 0.92
Deceased husband’s HIV status					
Negative	Ref	Ref	Ref	Ref	Ref
Positive	0.46 (0.22–0.97) 0.04, 0.28	0.44 (0.21–0.92) 0.03, 0.22	0.49 (0.24–1.03) 0.06	2.56 (0.89–7.36) 0.08, 0.36	0.85 (0.46–1.58) 0.61, 0.85
Don’t Know	1.05 (0.50–2.22) 0.89, 0.96	1.06 (0.50–2.23) 0.88, 0.95	1.06 (0.51–2.24) 0.87	0.45 (0.10–2.01) 0.30, 0.64	0.68 (0.37–1.26) 0.22, 0.58
Widow’s HIV status					
Negative	Ref	Ref	Ref	Ref	Ref
Positive	3.38 (1.76–6.49) <0.01, 0.01	3.22 (1.69–6.15) <0.01, 0.01	2.90 (1.53–5.51) <0.01	1.21 (0.44–3.32) 0.71, 0.90	1.60 (0.95–2.69) 0.08, 0.36

AOR = Adjusted Odds Ratio, CI = Confidence Interval; *p* = raw *p*-values that are unadjusted for multiple testing; q = false discovery rate adjusted *p*-values were calculated using Benjamini-Hochberg procedure; †Marital relatives: children, other family members or persons in the community; ††Includes those who reported no income activity or reported relying on support from family or government for means of livelihood.

For secondary outcomes, women widowed at a young age of <30 years (AOR: 34.5, 95%CI: 9.0–133.0, *p* < 0.001, q < 0.001) and 30–49 years (AOR: 19.0, 95%CI: 6.1–59.4, *p* < 0.001, q < 0.001) had higher odds of engaging in culturally sanctioned sexual practices of widow inheritance than women widowed when older (≥ 60 years) (Table 4). Similarly, women widowed at the age of <30 years (AOR: 41.5, 95%CI: 10.1–171.0, *p* < 0.001, q < 0.001) and 30–49 years (AOR: 25.4, 95%CI: 7.4–86.7, *p* < 0.001, q < 0.001) had higher odds of engaging in sexual cleansing than those widowed when above 60 years. Still, women widowed at an age of <30 years (AOR: 227.4, 95%CI: 12.0–4324.5, *p* < 0.001, q = 0.01) and 30–49 years (AOR: 106.9, 95%CI: 6.0–1917.2, *p* < 0.001, q = 0.04) had higher odds of engaging in intergenerational sex with partners 10 years or older than those widowed when above 60 years. Compared to HIV‑negative widows, widowed women living with HIV also had higher odds of engaging in widow inheritance (AOR: 3.4, 95%CI: 1.8–6.5, *p* < 0.001, q = 0.01) and sexual cleansing (AOR: 3.2, 95%CI: 1.7–6.2, *p* < 0.001, q = 0.01)

**Table 4.** Multivariable association between socio-demographic characteristics and HIV risk behaviors among widows recruited from a high-HIV prevalence setting in Kenya.

	Multiple sexual partnerships			Intimate partner violence	
	Inheritor had 2+ concurrent widows *N* = 438	Widow had 2+ sequential inheritors *N* = 471	Inheritor had own wife *N* = 438	Ever had forced sex *N* = 471	Stayed in abusive relationship *N* = 471
Variables	AOR (95% CI) p, q	AOR (95% CI) p, q	AOR (95% CI) p, q	AOR (95% CI) p, q	AOR (95% CI) p, q
Age at marriage, years					
≤24	Ref	Ref	Ref	Ref	Ref
25–30	1.77 (0.45–6.88) 0.41, 0.72	1.01 (0.34–3.04) 0.98, 0.99	0.94 (0.40–2.21) 0.89, 0.96	0.24 (0.03–1.86) 0.17, 0.54	7.15 (0.31–165.78) 0.22, 0.58
>30	3.36 (1.02–11.13) 0.05, 0.28	1.56 (0.60–4.07) 0.37, 0.69	1.03 (0.48–2.18) 0.95, 0.97	0.89 (0.20–4.00) 0.88, 0.95	2.49 (0.11–55.71) 0.57, 0.85
Age at widowhood, years					
<30	22.13 (1.09–451.19) 0.04, 0.27	8.37 (1.38–50.54) 0.02, 0.20	16.78 (2.79–100.84) <0.01, 0.05	19.85 (0.64–619.01) 0.09, 0.39	9.39 (0.25–350.35) 0.23, 0.59
30–49	15.77 (0.83–301.42) 0.07, 0.34	4.15 (0.74–23.32) 0.11, 0.42	16.73 (3.03–92.23) <0.01, 0.03	8.73 (0.34–225.01) 0.19, 0.57	8.59 (0.33–223.31) 0.20, 0.57
50–59	14.44 (0.75–278.81) 0.08, 0.36	0.57 (0.06–5.73) 0.63, 0.86	4.99 (0.84–29.49) 0.08, 0.36	2.28 (0.08–65.98) 0.63, 0.86	2.63 (0.07–103.36) 0.61, 0.85
≥60	Ref	Ref	Ref	Ref	Ref
Highest level of education attained					
None	Ref	Ref	Ref	Ref	Ref
Primary	0.87 (0.37–2.01) 0.74, 0.90	1.61 (0.65–4.01) 0.31, 0.65	1.59 (0.77–3.27) 0.21, 0.58	0.52 (0.15–1.78) 0.30, 0.64	0.22 (0.03–1.41) 0.11, 0.43
Secondary & above	0.13 (0.02–0.80) 0.03, 0.22	2.07 (0.68–6.32) 0.20, 0.57	1.81 (0.70–4.66) 0.22, 0.58	0.20 (0.03–1.31) 0.09, 0.40	0.52 (0.07–3.89) 0.52, 0.81
Completed vocational school					
No	Ref	Ref	Ref	Ref	Ref
Yes	0.82 (0.33–2.07) 0.68, 0.89	0.72 (0.33–1.58) 0.42, 0.72	0.59 (0.28–1.22) 0.15, 0.52	2.00 (0.64–6.21) 0.23, 0.59	1.66 (0.34–8.04) 0.53, 0.81
Head of household					
No	Ref	Ref	Ref	Ref	Ref
Yes	9.96 (0.47–211.01) 0.14, 0.51	6.44 (0.31–133.89) 0.23, 0.59	1.01 (0.25–4.14) 0.99, 0.99	0.22 (0.03–1.86) 0.17, 0.53	0.30 (0.04–2.03) 0.22, 0.58
Household size					
4 and below	Ref	Ref	Ref	Ref	Ref
Above 4	1.01 (0.54–1.86) 0.98, 0.99	1.22 (0.73–2.06) 0.44, 0.75	1.11 (0.69–1.79) 0.66, 0.88	0.79 (0.34–1.85) 0.59, 0.85	0.79 (0.22–2.86) 0.72, 0.91
Polygamous marriage with deceased husband					
No	Ref	Ref	Ref	Ref	Ref
Yes	0.65 (0.35–1.20) 0.17, 0.54	0.73 (0.43–1.25) 0.25, 0.62	1.56 (0.98–2.50) 0.06, 0.33	1.44 (0.61–3.42) 0.41, 0.72	1.05 (0.32–3.43) 0.94, 0.98
Christian denominations					
Catholic	Ref	Ref	Ref	Ref	Ref
African traditional churches	1.51 (0.56–4.05) 0.41, 0.72	0.97 (0.42–2.23) 0.94, 0.98	1.11 (0.53–2.31) 0.79, 0.93	2.43 (0.54–10.86) 0.25, 0.61	0.52 (0.09–3.14) 0.47, 0.78
Non-denominational churches	1.64 (0.64–4.22) 0.30, 0.65	0.98 (0.44–2.17) 0.97, 0.99	1.09 (0.53–2.24) 0.81, 0.93	1.71 (0.37–7.85) 0.49, 0.78	0.78 (0.14–4.45) 0.78, 0.93
Protestant	0.80 (0.28–2.25) 0.67, 0.88	0.82 (0.36–1.85) 0.63, 0.86	1.04 (0.50–2.18) 0.92, 0.97	1.52 (0.31–7.35) 0.61, 0.85	0.91 (0.15–5.44) 0.92, 0.97
Current economic activity					
Farming	Ref	Ref	Ref	Ref	Ref
Casual labor	1.62 (0.62–4.26) 0.33, 0.66	2.03 (0.94–4.37) 0.07, 0.35	0.47 (0.19–1.16) 0.10, 0.42	1.42 (0.43–4.77) 0.57, 0.85	2.81 (0.49–15.96) 0.24, 0.61
Employed	1.51 (0.06–35.24) 0.80, 0.92	0.27 (0.01–5.16) 0.39, 0.71	0.67 (0.09–5.02) 0.70, 0.90	1.18 (0.04–32.16) 0.92, 0.97	1.48 (0.04–55.81) 0.83, 0.94
Microbusiness	0.97 (0.50–1.88) 0.94, 0.98	0.72 (0.40–1.30) 0.28, 0.63	0.87 (0.53–1.42) 0.57, 0.84	1.08 (0.42–2.74) 0.88, 0.96	2.59 (0.60–11.08) 0.20, 0.57
Supported by others††	3.92 (1.24–12.41) 0.02, 0.21	1.11 (0.30–4.08) 0.88, 0.95	0.86 (0.30–2.47) 0.79, 0.93	0.47 (0.06–3.65) 0.47, 0.78	6.22 (0.90–43.21) 0.06, 0.33
Home ownership					
Yourself	Ref	Ref	Ref	Ref	Ref
Deceased spouse	1.48 (0.59–3.70) 0.40, 0.72	1.21 (0.57–2.57) 0.63, 0.86	1.02 (0.50–2.07) 0.95, 0.98	0.81 (0.21–3.19) 0.76, 0.92	2.29 (0.23–22.90) 0.48, 0.78
Marital relatives†	0.72 (0.26–2.00) 0.53, 0.81	0.79 (0.33–1.86) 0.58, 0.85	0.69 (0.31–1.55) 0.37, 0.69	9.13 (1.90–43.85) 0.01, 0.12	6.94 (0.56–85.53) 0.13, 0.49
Agricultural land ownership					
Yourself	Ref	Ref	Ref	Ref	Ref
Deceased spouse	1.09 (0.33–3.58) 0.89, 0.96	0.51 (0.20–1.30) 0.16, 0.53	0.42 (0.17–1.00) 0.05, 0.28	0.32 (0.08–1.26) 0.10, 0.42	0.57 (0.08–4.28) 0.59, 0.85
Marital relatives†	2.02 (0.62–6.65) 0.25, 0.61	0.72 (0.28–1.83) 0.49, 0.78	0.81 (0.34–1.93) 0.63, 0.86	0.18 (0.04–0.83) 0.03, 0.22	0.33 (0.04–3.05) 0.33, 0.66
Deceased husband’s HIV status					
Negative	Ref	Ref	Ref	Ref	Ref
Positive	1.18 (0.51–2.71) 0.70, 0.90	1.26 (0.63–2.55) 0.51, 0.80	0.67 (0.35–1.27) 0.22, 0.58	0.59 (0.18–1.97) 0.39, 0.71	0.42 (0.08–2.28) 0.31, 0.66
Don’t Know	1.11 (0.50–2.47) 0.79, 0.93	1.58 (0.80–3.12) 0.19, 0.56	0.74 (0.39–1.41) 0.36, 0.69	0.43 (0.14–1.36) 0.15, 0.52	0.71 (0.15–3.37) 0.67, 0.88
Widow’s HIV status					
Positive	Ref	Ref	Ref	Ref	Ref
Don’t Know	2.24 (1.10–4.56) 0.03, 0.22	1.57 (0.87–2.85) 0.14, 0.50	1.23 (0.72–2.13) 0.45, 0.76	1.65 (0.61–4.46) 0.33, 0.66	6.95 (1.42–34.08) 0.02, 0.21

AOR = Adjusted Odds Ratio, CI = Confidence Interval; *p* = raw *p*-values that are unadjusted for multiple testing; q = false discovery rate adjusted *p*-values were calculated using Benjamini-Hochberg procedure; †Marital relatives: children, other family members or persons in the community; ††Includes those who reported no income activity or reported relying on support from family or government for means of livelihood.

For multiple sexual partnerships, young widowhood of <30 years (AOR: 16.8, 95%CI: 2.8–100.8, *p* < 0.001, q = 0.05) and 30–49 years (AOR: 16.7, 95%CI: 3.0–92.2, *p* < 0.001, q = 0.03) was associated with widows engaging with inheritors who had their own wives (Table 4). Young widowhood, however, was not statistically significantly associated with intimate partner violence. In addition, young widowhood was not statistically significantly associated with different kinds of transactional sex or sex under the influence of drugs (Table 5).

**Table 5.** Multivariable association between socio-demographic characteristics and HIV risk behaviors among widows recruited from a high-HIV prevalence setting in Kenya.

	Transactional sex			Sex under influence of drugs *N* = 471
	Widow gave gifts for sex *N* = 471	Widow received gifts for sex *N* = 471	Sex work *N* = 471	
Variables	AOR (95% CI) p, q	AOR (95% CI) p, q	AOR (95% CI) p, q	AOR (95% CI) p, q
Age at marriage, years				
≤24	Ref	Ref	Ref	Ref
25–30	1.15 (0.35–3.84) 0.82, 0.93	0.84 (0.30–2.33) 0.74, 0.91	7.56 (0.40–142.90) 0.18, 0.55	2.56 (0.36–17.99) 0.35, 0.67
>30	0.64 (0.21–1.92) 0.42, 0.73	0.60 (0.24–1.52) 0.29, 0.63	8.36 (0.48–146.42) 0.15, 0.51	4.86 (0.81–29.26) 0.08, 0.38
Age at widowhood, years				
<30	11.86 (0.60–235.97) 0.10, 0.42	28.04 (1.52–516.35) 0.02, 0.22	28.52 (1.36–596.83) 0.03, 0.22	4.02 (0.52–31.12) 0.18, 0.56
30–49	12.17 (0.67–220.96) 0.09, 0.40	14.27 (0.82–246.93) 0.07, 0.34	9.00 (0.46–175.22) 0.15, 0.51	2.42 (0.37–15.89) 0.36, 0.69
50–59	2.86 (0.13–63.47) 0.51, 0.80	5.38 (0.28–103.76) 0.26, 0.62	5.92 (0.26–132.56) 0.26, 0.62	2.12 (0.29–15.66) 0.46, 0.77
≥60	Ref	Ref	Ref	Ref
Highest level of education attained				
None	Ref	Ref	Ref	Ref
Primary	1.56 (0.49–5.01) 0.45, 0.76	2.66 (0.78–9.02) 0.12, 0.44	0.79 (0.25–2.52) 0.69, 0.90	0.35 (0.13–0.98) 0.05, 0.28
Secondary & above	1.51 (0.35–6.51) 0.58, 0.85	2.73 (0.66–11.24) 0.16, 0.53	0.58 (0.13–2.63) 0.48, 0.78	0.22 (0.05–1.03) 0.05, 0.30
Completed vocational school				
No	Ref	Ref	Ref	Ref
Yes	0.80 (0.27–2.40) 0.70, 0.90	1.10 (0.46–2.59) 0.83, 0.94	0.87 (0.26–2.91) 0.82, 0.94	2.09 (0.75–5.85) 0.16, 0.53
Head of household				
No	Ref	Ref	Ref	Ref
Yes	1.48 (0.19–11.58) 0.71, 0.91	1.34 (0.19–9.38) 0.77, 0.92	0.36 (0.06–2.17) 0.27, 0.62	0.30 (0.05–1.91) 0.20, 0.57
Household size				
4 and below	Ref	Ref	Ref	Ref
Above 4	0.85 (0.42–1.72) 0.65, 0.87	1.25 (0.70–2.24) 0.45, 0.76	1.07 (0.50–2.29) 0.86, 0.96	0.86 (0.37–2.00) 0.73, 0.90
Polygamous marriage with deceased husband				
No	Ref	Ref	Ref	Ref
Yes	1.41 (0.70–2.83) 0.34, 0.66	1.48 (0.81–2.70) 0.20, 0.57	1.90 (0.88–4.11) 0.10, 0.42	1.55 (0.70–3.47) 0.28, 0.63
Christian denominations				
Catholic	Ref	Ref	Ref	Ref
African traditional churches	0.92 (0.33–2.53) 0.87, 0.96	0.71 (0.30–1.68) 0.43, 0.73	0.36 (0.12–1.06) 0.06, 0.33	0.74 (0.25–2.22) 0.59, 0.85
Non-denominational churches	1.00 (0.37–2.65) 0.99, 0.99	0.87 (0.38–1.96) 0.73, 0.90	0.54 (0.20–1.43) 0.21, 0.58	0.54 (0.18–1.61) 0.27, 0.62
Protestant	0.36 (0.11–1.18) 0.09, 0.40	0.42 (0.16–1.07) 0.07, 0.35	0.24 (0.07–0.78) 0.02, 0.21	0.44 (0.13–1.48) 0.18, 0.56
Current economic activity				
Farming	Ref	Ref	Ref	Ref
Casual labor	2.72 (1.00–7.44) 0.05, 0.29	1.28 (0.54–3.05) 0.58, 0.85	1.43 (0.49–4.15) 0.51, 0.80	3.67 (1.24–10.90) 0.02, 0.21
Employed	1.37 (0.07–28.74) 0.84, 0.95	0.62 (0.03–12.03) 0.75, 0.91	1.54 (0.07–34.57) 0.79, 0.93	2.73 (0.12–60.92) 0.53, 0.81
Microbusiness	2.72 (1.30–5.69) 0.01, 0.14	1.11 (0.59–2.08) 0.74, 0.90	0.69 (0.30–1.60) 0.39, 0.71	1.08 (0.44–2.60) 0.87, 0.96
Supported by others††	2.53 (0.50–12.66) 0.26, 0.62	1.05 (0.24–4.63) 0.95, 0.97	0.61 (0.12–3.19) 0.56, 0.84	1.01 (0.22–4.70) 0.99, 0.99
Home ownership				
Yourself	Ref	Ref	Ref	Ref
Deceased spouse	0.58 (0.20–1.68) 0.31, 0.65	0.67 (0.27–1.64) 0.38, 0.70	0.54 (0.18–1.64) 0.28, 0.63	0.42 (0.14–1.21) 0.11, 0.42
Marital relatives†	1.92 (0.60–6.16) 0.27, 0.63	1.86 (0.69–4.98) 0.22, 0.58	1.57 (0.47–5.20) 0.46, 0.77	0.41 (0.11–1.57) 0.19, 0.57
Agricultural land ownership				
Yourself	Ref	Ref	Ref	Ref
Deceased spouse	0.55 (0.17–1.79) 0.32, 0.66	1.00 (0.34–2.95) 0.99, 0.99	0.58 (0.18–1.90) 0.37, 0.69	1.16 (0.34–3.91) 0.82, 0.93
Marital relatives†	0.32 (0.09–1.11) 0.07, 0.35	0.57 (0.18–1.75) 0.32, 0.66	0.27 (0.08–0.97) 0.04, 0.27	0.57 (0.16–2.06) 0.40, 0.71
Deceased husband’s HIV status				
Negative	Ref	Ref	Ref	Ref
Positive	1.29 (0.49–3.39) 0.60, 0.85	1.12 (0.50–2.51) 0.78, 0.93	0.83 (0.28–2.41) 0.73, 0.91	1.71 (0.59–4.90) 0.32, 0.66
Don’t Know	3.20 (1.39–7.36) 0.01, 0.13	1.57 (0.74–3.30) 0.24, 0.60	1.45 (0.59–3.55) 0.42, 0.72	1.32 (0.51–3.44) 0.57, 0.85
Widow’s HIV status				
Negative	Ref	Ref	Ref	Ref
Positive	0.77 (0.35–1.68) 0.51, 0.80	0.59 (0.30–1.18) 0.14, 0.50	0.88 (0.38–2.05) 0.78, 0.93	1.36 (0.57–3.25) 0.49, 0.78

AOR = Adjusted Odds Ratio, CI = Confidence Interval; *p* = raw *p*-values that are unadjusted for multiple testing; q = false discovery rate adjusted *p*-values were calculated using Benjamini-Hochberg procedure; †Marital relatives: children, other family members or persons in the community; ††Includes those who reported no income activity or reported relying on support from family or government for means of livelihood.

## Discussion

In this manuscript, we address an important public health issue in a vulnerable and neglected population of women who have been featured less in quantitative research [29]. We provide an estimate of the likely period of HIV acquisition among widowed women living with HIV, the prevalence of HIV risk behaviors since widowhood, and the factors associated with the HIV risk behaviors in Gem sub-County, Siaya Kenya.

### HIV risk in widowhood

At least one in three of widowed women living with HIV may have acquired HIV during widowhood, as their time of HIV diagnosis was estimated at 5 years after the start of widowhood. While much literature has shown a high HIV prevalence among widowed women [1–7], little data exist on the timing of HIV acquisition (during marriage or during widowhood). The majority of studies have assumed HIV infection of the spouse to be the cause of widowhood [18,19]. Yet at least a third of the widowed women with HIV in our study may have been infected after widowhood. While the evidence we provide on the risk of HIV acquisition in widowhood is not conclusive, this is one of the first studies to describe HIV exposure during widowhood. Future research should include HIV recency tests or longitudinal testing to more accurately determine the magnitude of HIV acquisition in widowhood.

The most common HIV risk behaviors in widowhood were culturally sanctioned sexual practices of widow inheritance and sexual cleansing, which were highly correlated with condomless sex. Previous work has shown the prohibition of use in these culturally sanctioned sexual practices and its association with the acquisition and or transmission of HIV for widows and their sexual partners [11,24,34,35]. For our study, at least a third of the widowed women living with HIV may have been exposed through these practices. The cultural requirement for condomless sex in a high HIV prevalence setting is a form of sexual exploitation [34]. HIV prevention services targeting widowed women and their appropriate delivery methods should be studied further.

### Risk factors for HIV risk behaviors in widowhood

Young widowhood was associated with five of the 14 studied HIV risk behaviors, including condomless sex, widow inheritance, and sexual cleansing. While older widowed women can use their age to negotiate out of culturally sanctioned sexual practices [36], younger widowed women not only have less liberty to do so, but may also desire remarriage, children, or a father figure for their children [31]. In addition, young widowhood was associated with multiple sexual partnerships in which widow inheritors had their own wives. Widow remarriage in the study context was limited to the practice of widow inheritance, that is, polygamous and temporary by design, exposing widows to a lack of full remarriage rights and unstable sexual relationships [11,12,14,17,30,31,37]. Thus, alternative remarriage practices for widowed women in this cultural context should be explored to ensure young widows can access stable relationships that reduce HIV risk behaviors.

Young widowhood was also associated with intergenerational sex with older partners. Intergenerational sex has been well studied among adolescent girls and young women with older men and shown to increase HIV risk [38–41]. A qualitative study in Kenya described intergenerational sex between older widows and young men [42] with a recent quantitative study showing higher odds of HIV prevalence among widows in such relationships [24]. Our study found a low prevalence of widows partnering intergenerationally with younger sexual partners. The directionality of HIV risk in these age-disparate relationships needs further investigation.

Living with HIV among widows was associated with culturally sanctioned sexual practices such as condomless sex, widow inheritance, and sexual cleansing. Past studies in SSA have described widows as HIV transmitters through practices of sexual cleansing and widow inheritance [11,17,43–45]. Due to the changing nature of these culturally sanctioned sexual practices—for example, the shift from relatives as widow inheritors to non-relatives as professional widow inheritors—there is a potential for increased risk of new HIV infection or re-infections for widows [11,12,46–48]. Widows living with HIV also face additional vulnerabilities that limit their ability to resist these cultural practices, including the fear of additional stigma and discrimination and economic vulnerabilities [14,29,30]. Thus, their participation in culturally sanctioned sexual practices can also be a means of accessing family acceptance and support [34,49]. While we cannot determine what came first—HIV or the risk behaviors—these complex, multi-directional risk webs are important to understand to inform HIV prevention strategies in this population.

In our study, late marriage (after 30 years of age) was associated with condomless sex during widowhood. Most marriage studies in sub-Saharan Africa (SSA) have focused on the determinants and effects of early marriage [50–52]. Few older studies have found late marriage associated with HIV risk, mediated through a longer premarital period of exposure to multiple and changing sexual partners [53–55]. In our study, late marriage was associated with a shorter duration of marriage, which may have led to condomless sex in attempts to bear children and continue the deceased husband’s lineage [56].

All the widows in our study identified with different denominations of Christianity, and belonging to African Traditional Churches was associated with condomless sex. African Traditional Churches tend to promote the integration of Christian values with African culture, which make them more permissive in the practice of culturally sanctioned sexual practices that require condomless sex [57]. Religious leaders in African Traditional Churches, in contexts of high HIV prevalence such as the study setting, need a critical view of culture and theology. There is a need to advance aspects of culture that promote the health and wellbeing of widowed women, while providing alternatives to aspects that increase HIV risk among widows [35,58].

Consistent with prior literature, vocational education was a protective factor against condomless sex among widowed women. Our findings affirmed the role of vocational education in the long-term empowerment and protection of women against HIV‑risk behaviours [59,60]. Importantly, vocational schools should not only target students transitioning out of secondary school, but also young widows with only primary education or without formal education at all, to reduce their engagement in HIV‑risk behaviors.

Contrary to expectation, we found a low prevalence of transactional sex and intimate partner violence, and no socio-demographic or economic factors were significantly associated with either. Several qualitative studies have discussed the participation of widows in transactional sex due to heightened financial insecurities [13,61,62]. Widowed women also report experiences of forced sex due to social, cultural, and economic contexts [23,34]. Given the sensitivities surrounding these HIV risk behaviours, our study may have under-reported these experiences. Still, it is possible that these experiences have been captured in prior qualitative work due to their intensities, but that their prevalences are low. Interventions to address safety and economic insecurities should be targeted to widows exposed to various forms of transactional sex and or intimate partner violence.

Similarly, polygamous marriage, partner HIV status, household size, economic activity, and asset ownership—variables that previous studies have associated with HIV risk behaviours—were not associated with the outcomes in our study [63–65]. This is likely due to the effect of confounding where the crude effect was attenuated after controlling for other covariates. It is also possible that our study was underpowered to detect the effect of these variables. Some of the predictors, such as polygamous marriage, have also yielded mixed results in different settings and require contextual understanding.

### Limitations

There are several limitations to this study. This was an exploratory study of HIV risk behaviors and risk factors among widows in Siaya County, Kenya. Firstly, while we indicate a proportion of widowed women who may have acquired HIV during widowhood, we lacked HIV recency tests for confirmation. We used sensitivity analyses of two to 5 years between the time of HIV diagnosis and the estimated time of HIV exposure. Future studies should include recency tests or longitudinal testing to better determine the magnitude of HIV infection during widowhood.

Secondly, a key limitation of this study was the temporal ambiguity between exposure measures and HIV risk behaviors. We measured HIV risk behaviors since widowhood with large time periods ranging ≤ 2 years to >20 years, indicating that the risk behaviors may have occurred at any time in large window period. In contrast , predictors such as age, economic activity, and marital status were measured at the time of survey. Given the cross-sectional design of our study, this limited our ability to establish temporal ordering or infer causal pathways. The long periods for measuring HIV risk behaviors increased the likelihood of recall bias, especially for women widowed a long time ago. Future studies can sample sexually active widowed women or recently widowed women and include longitudinal designs to assess the most current risk behaviors and the motivations behind those risk behaviors.

Several outcomes, such as transactional sex, captured sensitive sexual behaviors that may have been underreported due to social desirability bias. This may have biased results toward the null, leading to an underestimation of potentially true associations and missing important risk factors. A few of the outcomes we measured also needed more nuanced definitions. For example, the measure of multiple sexual partnerships focused primarily on widow inheritance, yet there are widowed women who may not consider their sexual partners as widow inheritors. Thus, we likely underestimated the effect of multiple sexual partnerships on HIV risk behaviors. For the outcome of intimate partner violence, we did not disaggregate it to its components of sexual, physical, and emotional violence, which would provide a nuanced understanding of the nature of abuse; this should be addressed in future analyses. Because the majority of the data, such as the deceased husband’s HIV status, were self-reported by the widows, there was a potential for general measurement error.

Despite using penalized regression, a few predictors still yielded very large odds ratios or wide confidence intervals. In addition, we observed wide confidence intervals for some outcomes, such as intimate partner violence. While this may reflect small sample sizes and sparse data in certain categories, the direction and magnitude of the association of predictors such as young widowhood were consistent across multiple outcomes, suggesting that the observed relationship was not purely a statistical artifact and may reflect a meaningful underlying effect. Still, these estimates with wide confidence intervals should be interpreted with caution, as they reduce the strength of inferences drawn. Emphasis should be placed on the direction of effects rather than statistical significance.

Finally, our study took place in a single sub-county in Siaya, Kenya, with a specific cultural context, limiting generalizability to similar contexts. In addition, due to the time gap between when the sampling frame was developed in 2022, and when we collected data in 2024, widows who died prior to data collection and women who became widowed after the creation of the sampling frame were not represented in our study frame, introducing selection bias that may limit the generalizability of our results.

## Conclusions

One in three widowed women with HIV may have acquired the virus during widowhood. HIV testing, pre-exposure prophylaxis and the use of condoms in culturally sanctioned sexual practices should be promoted and studied further. Given the association of young widowhood with multiple HIV‑risk behaviors, alternative remarriage practices for widowed women may reduce their HIV risk behaviors. In addition, vocational education may reduce widows’ engagement in HIV‑risk behaviors.

## Supplementary Material

SUPPLEMENTARY MATERIALS_v3_clean.docx

GHA STROBE crossectional.docx

## Data Availability

The data that support the findings of this study are available from the authors upon reasonable request.
