# Supplementary material for: Young widowhood as a predictor of HIV risk behaviors in a high HIV prevalence setting in Siaya County, Kenya
Source: Glob Health Action. 2026 Jun 17;19(1):2684829. doi: 10.1080/16549716.2026.2684829 (PMC13276819; doi:10.1080/16549716.2026.2684829)
Supplement: SUPPLEMENTARY MATERIALS_v3_clean.docx [file ZGHA_A_2684829_SM7025.docx]

**SUPPLEMENTARY MATERIALS**

**Supplementary Table 1a. Unadjusted association between socio-demographic characteristics and HIV risk behaviors among widows recruited from a high-HIV prevalence setting in Kenya**

|  | **Culturally sanctioned sexual practices** | | | **Intergenerational sex** | |
| --- | --- | --- | --- | --- | --- |
|  | **Widow inheritance** | **Sexual cleansing** | **Primary Outcome**  **Condomless sex** | **10+ year younger sex partner** | **10+ year older sex partner** |
| **Variables** | **AOR (95% CI) p, q** | **AOR (95% CI) p, q** | **AOR (95% CI) p** | **AOR (95% CI) p, q** | **AOR (95% CI) p, q** |
| **Age at marriage, years** |  |  |  |  |  |
| ≤24 | Ref | Ref | Ref | Ref | Ref |
| 25-30 | 1.20 (0.65–2.21) 0.57, 0.76 | 1.15 (0.62–2.12) 0.66, 0.82 | 1.32 (0.71–2.44) 0.38 | 1.34 (0.17–10.40) 0.78, 0.89 | 1.37 (0.68–2.78) 0.38, 0.62 |
| >30 | **4.16 (2.47–7.03) <0.01, <0.01** | **4.16 (2.47–7.03) <0.01, <0.01** | **4.33 (2.56–7.32) <0.01** | 4.22 (0.79–22.43) 0.09, 0.27 | **2.46 (1.36–4.46) <0.01, 0.02** |
| **Age at widowhood, years** |  |  |  |  |  |
| <30 | **88.39 (28.21–276.97) <0.01, <0.01** | **105.26 (30.67–361.30) <0.01, <0.01** | **143.42 (40.22–511.40) <0.01** | 15.47 (0.89–267.71) 0.06, 0.08 | **157.59 (9.46–2626.09) <0.01, <0.01** |
| 30-49 | **29.44 (10.83–80.01) <0.01, <0.01** | **39.31 (12.89–119.87) <0.01, <0.01** | **37.10 (12.18–113.04) <0.01** | 7.63 (0.45–129.48) 0.16, 0.20 | **77.85 (4.75–1274.69) <0.01, 0.01** |
| 50-59 | **4.73 (1.59–14.07) 0.01, 0.01** | **6.20 (1.87–20.49) <0.01, 0.01** | **6.20 (1.87–20.49) <0.01** | 0.82 (0.02–41.75) 0.92, 0.92 | 20.29 (1.16–354.43) 0.04, 0.06 |
| ≥60 | Ref | Ref | Ref | Ref | **Ref** |
| **Highest level of education attained** | |  |  |  |  |
| None | Ref | Ref | Ref | Ref | Ref |
| Primary | **2.40 (1.50–3.83) <0.01, <0.01** | **2.37 (1.49–3.78) <0.01, <0.01** | **2.47 (1.55–3.95) <0.01** | 12.85 (0.77–214.21) 0.08, 0.24 | 1.68 (1.00–2.81) 0.05, 0.18 |
| Secondary & above | 1.29 (0.66–2.52) 0.46, 0.69 | 1.29 (0.66–2.52) 0.46, 0.69 | 1.45 (0.74–2.84) 0.28 | 20.98 (1.14–387.40) 0.04, 0.16 | 0.98 (0.46–2.10) 0.96, 0.97 |
| **Completed vocational school** |  |  |  |  |  |
| No | Ref | Ref | Ref | Ref | Ref |
| Yes | 0.58 (0.33–1.01) 0.05, 0.18 | 0.58 (0.33–1.02) 0.06, 0.20 | 0.63 (0.36–1.10) 0.10 | 1.55 (0.54–4.44) 0.42, 0.65 | 0.60 (0.32–1.12) 0.11, 0.29 |
| **Head of household** |  |  |  |  |  |
| No | Ref | Ref | Ref | Ref | Ref |
| Yes | 2.40 (0.94–6.14) 0.07, 0.22 | 2.38 (0.93–6.08) 0.07, 0.22 | 1.90 (0.75–4.78) 0.17 | 2.27 (0.13–38.75) 0.57, 0.77 | 2.46 (0.76–7.98) 0.13, 0.31 |
| **Household size** |  |  |  |  |  |
| 4 and below | Ref | Ref | Ref | Ref | Ref |
| Above 4 | **1.77 (1.18–2.65) 0.01, 0.04** | **1.87 (1.24–2.80) <0.01, 0.02** | **1.79 (1.19–2.68) <0.01** | 1.16 (0.51–2.62) 0.72, 0.86 | **2.00 (1.34–2.99) <0.01, 0.01** |
| **Polygamous marriage with deceased husband** | |  |  |  |  |
| No | Ref | Ref | Ref | Ref | Ref |
| Yes | 0.67 (0.47–0.97) 0.04, 0.15 | 0.69 (0.48–0.99) 0.04, 0.17 | **0.69 (0.48–0.99) 0.04** | 0.81 (0.37–1.76) 0.59, 0.75 | 0.83 (0.57–1.22) 0.34, 0.59 |
| **Christian denominations** |  |  |  |  |  |
| Catholic | Ref | Ref | Ref | Ref | Ref |
| African traditional churches | **2.67 (1.48–4.80) <0.01, 0.01** | **2.48 (1.39–4.45) <0.01, 0.02** | **2.57 (1.43–4.62) <0.01** | 0.46 (0.14–1.50) 0.20, 0.40 | 1.51 (0.83–2.76) 0.18, 0.37 |
| Non-denominational churches | 1.58 (0.90–2.76) 0.11, 0.28 | 1.58 (0.90–2.76) 0.11, 0.28 | 1.58 (0.90–2.76) 0.11 | 0.67 (0.23–1.92) 0.45, 0.68 | 1.05 (0.58–1.92) 0.87, 0.93 |
| Protestant | 1.18 (0.67–2.09) 0.56, 0.76 | 1.22 (0.69–2.16) 0.49, 0.70 | 1.18 (0.67–2.09) 0.56 | 0.68 (0.23–2.03) 0.49, 0.70 | 1.25 (0.68–2.30) 0.47, 0.70 |
| **Current economic activity** |  |  |  |  |  |
| Farming | Ref | Ref | Ref | Ref | Ref |
| Casual labor | 1.30 (0.66–2.57) 0.44, 0.69 | 1.28 (0.65–2.53) 0.48, 0.72 | 1.20 (0.61–2.34) 0.60 | **4.05 (1.51–10.83) 0.01, 0.02** | 1.85 (0.96–3.53) 0.06, 0.16 |
| Employed | 0.90 (0.17–4.64) 0.90, 0.94 | 0.88 (0.17–4.56) 0.88, 0.95 | 0.91 (0.18–4.72) 0.91 | 6.57 (0.94–45.71) 0.06, 0.15 | 0.64 (0.10–4.17) 0.64, 0.90 |
| Microbusiness | 1.29 (0.83–1.99) 0.26, 0.44 | 1.19 (0.77–1.83) 0.44, 0.69 | 1.35 (0.87–2.10) 0.17 | 1.12 (0.44–2.89) 0.81, 0.95 | 1.14 (0.73–1.77) 0.56, 0.81 |
| Supported by others†† | **0.32 (0.17–0.59) <0.01, <0.01** | **0.31 (0.17–0.58) <0.01, <0.01** | **0.32 (0.17–0.60) <0.01** | 0.19 (0.01–3.23) 0.25, 0.44 | 0.61 (0.31–1.22) 0.16, 0.32 |
| **Home ownership** |  |  |  |  |  |
| Yourself | Ref | Ref | Ref | Ref | Ref |
| Deceased spouse | **0.30 (0.16–0.53) <0.01, <0.01** | **0.32 (0.18–0.57) <0.01, <0.01** | **0.32 (0.18–0.56) <0.01** | **0.28 (0.11–0.68) 0.01, 0.03** | 0.71 (0.42–1.20) 0.20, 0.40 |
| Marital relatives† | 0.72 (0.36–1.43) 0.35, 0.59 | 0.77 (0.39–1.53) 0.46, 0.69 | 0.81 (0.41–1.62) 0.55 | 0.48 (0.17–1.37) 0.17, 0.36 | 1.12 (0.61–2.06) 0.71, 0.86 |
| **Agricultural land ownership** |  |  |  |  |  |
| Yourself | Ref | Ref | Ref | Ref | Ref |
| Deceased spouse | **0.41 (0.21–0.78) 0.01, 0.04** | 0.44 (0.23–0.85) 0.01, 0.07 | **0.40 (0.21–0.77) 0.01** | 0.40 (0.14–1.16) 0.09, 0.27 | 0.65 (0.35–1.22) 0.18, 0.37 |
| Marital relatives† | 0.90 (0.45–1.80) 0.78, 0.89 | 0.99 (0.50–1.96) 0.99, 0.99 | 0.90 (0.45–1.80) 0.78 | 0.55 (0.19–1.63) 0.28, 0.51 | 1.24 (0.65–2.36) 0.51, 0.71 |
| **Deceased husband’s HIV status** |  |  |  |  |  |
| Negative | Ref | Ref | Ref | Ref | Ref |
| Positive | 1.70 (1.07–2.71) 0.02, 0.11 | 1.63 (1.03–2.60) 0.04, 0.15 | **1.73 (1.09–2.75) 0.02** | **3.47 (1.53–7.89) <0.01, 0.02** | 1.62 (1.02–2.56) 0.04, 0.16 |
| Don't Know | **2.36 (1.39–3.99) <0.01, 0.01** | **2.36 (1.39–3.99) <0.01, 0.01** | **2.39 (1.41–4.04) <0.01** | 0.70 (0.18–2.82) 0.62, 0.78 | 1.23 (0.74–2.04) 0.43, 0.66 |
| **Widows’ HIV status** |  |  |  |  |  |
| Negative | Ref | Ref | Ref | Ref | Ref |
| Positive | **4.12 (2.68–6.31) <0.01, <0.01** | **3.98 (2.60–6.08) <0.01, <0.01** | **3.98 (2.60–6.08) <0.01** | **2.81 (1.26–6.23) 0.01, 0.02** | **2.18 (1.48–3.23) <0.01, <0.01** |
|  |  |  |  |  |  |
| AOR = Adjusted Odds Ratio, CI = Confidence Interval; p = raw p-values that are unadjusted for multiple testing; q = false discovery rate adjusted p-values were calculated using Benjamini-Hochberg procedure; †Marital relatives: children, other family members or persons in the community; ††Includes those who reported no income activity or reported relying on support from family or government for means of livelihood | | | | | |

**Supplementary Table 1b. Unadjusted association between socio-demographic characteristics and HIV risk behaviors among widows recruited from a high-HIV prevalence setting in Kenya**

|  | | **Multiple sexual partnerships** | | | | **Intimate partner violence** | | |
| --- | --- | --- | --- | --- | --- | --- | --- | --- |
|  | | **Inheritor had 2+ concurrent widows** | **Widow had 2+ sequential inheritors** | **Inheritor had own wife** | | **Ever had forced sex** | **Stayed in abusive relationship** | |
| **Variables** | | **AOR (95% CI) p, q** | **AOR (95% CI) p, q** | **AOR (95% CI) p, q** | | **AOR (95% CI) p, q** | **AOR (95% CI) p, q** | |
| **Age at marriage, years** | |  |  |  | |  |  | |
| ≤24 | | Ref | Ref | Ref | | Ref | Ref | |
| 25-30 | | 1.74 (0.47–6.43) 0.41, 0.64 | 1.49 (0.54–4.11) 0.44, 0.67 | 1.24 (0.58–2.68) 0.58, 0.75 | | 0.81 (0.14–4.77) 0.81, 0.91 | 7.61 (0.40–143.57) 0.18, 0.37 | |
| >30 | | **4.56 (1.49–13.90) 0.01, 0.05** | **3.54 (1.52–8.23) <0.01, 0.03** | 1.96 (1.03–3.73) 0.04, 0.16 | | 2.97 (0.79–11.13) 0.11, 0.29 | 5.49 (0.32–94.78) 0.24, 0.45 | |
| **Age at widowhood, years** | |  |  |  | |  |  | |
| <30 | | **36.41 (2.15–616.10) 0.01, 0.02** | **27.00 (5.08–143.56) <0.01, <0.01** | **19.00 (3.53–102.38) <0.01, <0.01** | | 21.87 (1.28–372.99) 0.03, 0.06 | 6.91 (0.38–127.38) 0.19, 0.23 | |
| 30-49 | | 19.75 (1.19–327.12) 0.04, 0.06 | **9.21 (1.77–47.99) 0.01, 0.02** | **19.29 (3.73–99.88) <0.01, <0.01** | | 7.54 (0.44–127.84) 0.16, 0.20 | 4.01 (0.23–70.57) 0.34, 0.39 | |
| 50-59 | | 12.91 (0.72–231.11) 0.08, 0.11 | 0.80 (0.08–7.91) 0.85, 0.87 | 6.20 (1.08–35.67) 0.04, 0.06 | | 2.45 (0.10–61.21) 0.59, 0.61 | 2.45 (0.10–61.21) 0.59, 0.61 | |
| ≥60 | | Ref | Ref | Ref | | Ref | Ref | |
| **Highest level of education attained** | | |  |  | |  |  | |
| None | Ref | | Ref | Ref | | Ref | Ref | |
| Primary | 1.31 (0.66–2.60) 0.45, 0.68 | | **3.08 (1.40–6.80) 0.01, 0.03** | 1.98 (1.09–3.60) 0.03, 0.12 | | 1.31 (0.50–3.38) 0.58, 0.75 | 0.51 (0.16–1.64) 0.26, 0.48 | |
| Secondary & above | 0.21 (0.04–1.19) 0.08, 0.24 | | **4.18 (1.60–10.92) <0.01, 0.03** | 2.13 (0.95–4.80) 0.07, 0.22 | | 0.77 (0.17–3.58) 0.74, 0.87 | 1.36 (0.32–5.75) 0.67, 0.82 | |
| **Completed vocational school** |  | |  |  | |  |  | |
| No | Ref | | Ref | Ref | | Ref | Ref | |
| Yes | 0.80 (0.34–1.90) 0.61, 0.77 | | 1.07 (0.53–2.12) 0.86, 0.93 | 0.76 (0.39–1.49) 0.43, 0.66 | | 1.94 (0.78–4.79) 0.15, 0.34 | 2.16 (0.64–7.31) 0.22, 0.41 | |
| **Head of household** |  | |  |  | |  |  | |
| No | Ref | | Ref | Ref | | Ref | Ref | |
| Yes | 6.29 (0.37–105.76) 0.20, 0.40 | | 9.24 (0.55–154.71) 0.12, 0.31 | 1.21 (0.41–3.56) 0.73, 0.86 | | 0.47 (0.12–1.85) 0.28, 0.50 | **0.12 (0.03–0.45) <0.01, 0.01** | |
| **Household size** |  | |  |  | |  |  | |
| 4 and below | Ref | | Ref | Ref | | Ref | Ref | |
| Above 4 | 1.08 (0.61–1.90) 0.80, 0.91 | | 1.49 (0.93–2.39) 0.09, 0.27 | 1.32 (0.85–2.06) 0.21, 0.41 | | 0.99 (0.47–2.12) 0.99, 0.99 | 0.83 (0.27–2.51) 0.74, 0.87 | |
| **Polygamous marriage with deceased husband** | | |  |  | |  |  | |
| No | | Ref | Ref | Ref | | Ref | Ref | |
| Yes | | 0.70 (0.41–1.20) 0.20, 0.40 | 0.60 (0.38–0.95) 0.03, 0.13 | 1.20 (0.79–1.83) 0.38, 0.62 | | 0.82 (0.40–1.66) 0.58, 0.76 | 1.06 (0.39–2.88) 0.91, 0.95 | |
| **Christian denominations** | |  |  |  | |  |  | |
| Catholic | | Ref | Ref | Ref | | Ref | Ref | |
| African traditional churches | | 1.71 (0.70–4.18) 0.24, 0.45 | 1.13 (0.55–2.34) 0.74, 0.87 | 1.41 (0.73–2.73) 0.30, 0.54 | | 3.27 (0.82–12.99) 0.09, 0.27 | 1.01 (0.21–4.88) 0.99, 0.99 | |
| Non-denominational churches | | 1.94 (0.81–4.64) 0.14, 0.32 | 1.22 (0.60–2.49) 0.58, 0.75 | 1.18 (0.61–2.27) 0.63, 0.79 | | 2.28 (0.56–9.32) 0.25, 0.47 | 1.15 (0.25–5.27) 0.86, 0.93 | |
| Protestant | | 0.95 (0.36–2.51) 0.92, 0.95 | 1.06 (0.51–2.22) 0.87, 0.93 | 1.12 (0.57–2.20) 0.75, 0.87 | | 1.86 (0.43–8.02) 0.40, 0.64 | 1.09 (0.23–5.25) 0.92, 0.95 | |
| **Current economic activity** | |  |  |  | |  |  | |
| Farming | | Ref | Ref | Ref | | Ref | Ref | |
| Casual labor | | 1.80 (0.73–4.40) 0.20, 0.37 | **2.70 (1.37–5.35) <0.01, 0.02** | 0.65 (0.27–1.52) 0.32, 0.53 | | 3.18 (1.16–8.73) 0.02, 0.07 | 4.10 (0.78–21.51) 0.09, 0.21 | |
| Employed | | 0.60 (0.03–11.12) 0.73, 0.99 | 0.37 (0.02–6.85) 0.51, 0.75 | 0.79 (0.12–5.12) 0.80, 0.96 | | 1.67 (0.09–31.91) 0.73, 0.95 | 6.19 (0.28–134.99) 0.25, 0.44 | |
| Microbusiness | | 1.11 (0.60–2.06) 0.73, 0.97 | 0.91 (0.53–1.56) 0.73, 0.95 | 1.02 (0.64–1.62) 0.95, 0.96 | | 1.80 (0.80–4.06) 0.16, 0.34 | 3.32 (0.89–12.38) 0.07, 0.17 | |
| Supported by others†† | | 1.11 (0.47–2.64) 0.81, 0.93 | 0.29 (0.09–0.90) 0.03, 0.09 | 0.40 (0.17–0.91) 0.03, 0.08 | | 0.91 (0.23–3.66) 0.89, 0.94 | **6.32 (1.51–26.44) 0.01, 0.04** | |
| **Home ownership** | |  |  |  | |  |  | |
| Yourself | | Ref | Ref | Ref | | Ref | Ref | |
| Deceased spouse | | 0.81 (0.39–1.71) 0.58, 0.75 | 0.52 (0.29–0.95) 0.03, 0.14 | 0.63 (0.36–1.11) 0.11, 0.28 | | 0.41 (0.14–1.22) 0.11, 0.29 | 1.25 (0.21–7.36) 0.80, 0.91 | |
| Marital relatives† | | 1.02 (0.44–2.40) 0.96, 0.97 | 0.81 (0.41–1.61) 0.55, 0.75 | 0.70 (0.36–1.36) 0.29, 0.52 | | 2.84 (1.04–7.75) 0.04, 0.16 | 3.94 (0.67–23.36) 0.13, 0.31 | |
| **Agricultural land ownership** | |  |  |  | |  |  | |
| Yourself | | Ref | Ref | Ref | | Ref | Ref | |
| Deceased spouse | | 0.90 (0.34–2.38) 0.83, 0.92 | 0.51 (0.25–1.04) 0.06, 0.21 | 0.52 (0.26–1.04) 0.06, 0.21 | | 0.27 (0.09–0.76) 0.01, 0.07 | 0.51 (0.11–2.26) 0.38, 0.62 | |
| Marital relatives† | | 1.52 (0.57–4.04) 0.40, 0.64 | 0.88 (0.43–1.79) 0.72, 0.86 | 0.83 (0.41–1.67) 0.60, 0.76 | | 0.79 (0.30–2.07) 0.64, 0.79 | 0.90 (0.21–3.91) 0.89, 0.94 | |
| **Deceased husband’s HIV status** | | |  |  | |  |  | |
| Negative | | Ref | Ref | Ref | | Ref | Ref | |
| Positive | | 1.66 (0.88–3.14) 0.12, 0.30 | **2.09 (1.21–3.60) 0.01, 0.05** | 1.21 (0.73–2.01) 0.47, 0.69 | | 1.53 (0.67–3.48) 0.31, 0.55 | 1.66 (0.51–5.47) 0.40, 0.64 | |
| Don't Know | | 1.69 (0.86–3.33) 0.13, 0.31 | **2.30 (1.30–4.07) <0.01, 0.03** | 1.03 (0.59–1.81) 0.92, 0.95 | | 1.24 (0.49–3.15) 0.65, 0.81 | 2.02 (0.61–6.68) 0.25, 0.46 | |
| **Widows’ HIV status** | |  |  |  |  | | |  |
| Negative | | Ref | Ref | Ref | Ref | | | Ref |
| Positive | | **2.54 (1.48–4.36) <0.01, <0.01** | **2.84 (1.79–4.52)** **<0.01, <0.01** | **1.56 (1.02–2.40) 0.04, 0.05** | **2.29 (1.12–4.68) 0.02, 0.03** | | | **3.37 (1.18–9.62) 0.02, 0.03** |
|  | |  |  |  |  | | |  |
| AOR = Adjusted Odds Ratio, CI = Confidence Interval; p = raw p-values that are unadjusted for multiple testing; q = false discovery rate adjusted p-values were calculated using Benjamini-Hochberg procedure; †Marital relatives: children, other family members or persons in the community; ††Includes those who reported no income activity or reported relying on support from family or government for means of livelihood | | | | | | | | |

**Supplementary Table 1c. Unadjusted association between socio-demographic characteristics and HIV risk behaviors among widows recruited from a high-HIV prevalence setting in Kenya**

|  | | **Transactional sex** | | | | | |  |  |  |
| --- | --- | --- | --- | --- | --- | --- | --- | --- | --- | --- |
|  | | **Widow gave gifts for sex** | | **Widow received gifts for sex** | | **Sex work** | | **Sex under influence of drugs** |  |  |
| **Variables** | | **AOR (95% CI) p, q** | | **AOR (95% CI) p, q** | | **AOR (95% CI) p, q** | | **AOR (95% CI) p, q** |  |  |
| **Age at marriage, years** | |  | |  | |  | |  |  |  |
| ≤24 | | Ref | | Ref | | Ref | | Ref |  |  |
| 25-30 | | 1.62 (0.55–4.75) 0.38, 0.62 | | 1.22 (0.48–3.09) 0.67, 0.82 | | 11.24 (0.62–202.75) 0.10, 0.29 | | 2.50 (0.38–16.29) 0.34, 0.58 |  |  |
| >30 | | 1.40 (0.54–3.61) 0.48, 0.70 | | 1.24 (0.57–2.73) 0.59, 0.75 | | 17.75 (1.07–293.46) 0.04, 0.17 | | 5.02 (0.95–26.50) 0.06, 0.20 |  |  |
| **Age at widowhood, years** | |  | |  | |  | |  |  |  |
| <30 | | 18.59 (1.08–319.07) 0.04, 0.07 | | **36.77 (2.19–618.30) 0.01, 0.02** | | **28.94 (1.71–489.27) 0.02, 0.03** | | 5.07 (0.89–28.70) 0.07, 0.09 |  |  |
| 30-49 | | 15.25 (0.92–253.32) 0.06, 0.08 | | 17.63 (1.06–292.13) 0.05, 0.07 | | 8.06 (0.48–136.30) 0.15, 0.19 | | 2.81 (0.52–15.27) 0.23, 0.27 |  |  |
| 50-59 | | 4.14 (0.19–87.91) 0.36, 0.40 | | 7.67 (0.40–145.44) 0.17, 0.21 | | 4.14 (0.19–87.91) 0.36, 0.40 | | 1.92 (0.28–13.46) 0.51, 0.55 |  |  |
| ≥60 | | Ref | | Ref | | Ref | | Ref |  |  |
| **Highest level of education attained** | | | |  | |  | |  |  |  |
| None | | Ref | | Ref | | Ref | | Ref |  |  |
| Primary | | 1.82 (0.72–4.62) 0.21, 0.41 | | **4.42 (1.46–13.40) 0.01, 0.05** | | 1.47 (0.57–3.79) 0.42, 0.65 | | 0.68 (0.30–1.56) 0.36, 0.60 |  |  |
| Secondary & above | | 1.80 (0.53–6.19) 0.35, 0.59 | | 4.78 (1.31–17.44) 0.02, 0.09 | | 1.45 (0.40–5.27) 0.58, 0.76 | | 0.69 (0.19–2.51) 0.57, 0.76 |  |  |
| **Completed vocational school** | |  | |  | |  | |  |  |  |
| No | | Ref | | Ref | | Ref | | Ref |  |  |
| Yes | | 0.81 (0.29–2.25) 0.69, 0.83 | | 1.05 (0.46–2.39) 0.90, 0.95 | | 0.74 (0.24–2.31) 0.60, 0.77 | | 1.54 (0.59–4.03) 0.38, 0.62 |  |  |
| **Head of household** | |  | |  | |  | |  |  |  |
| No | | Ref | | Ref | | Ref | | Ref |  |  |
| Yes | | 1.21 (0.22–6.60) 0.83, 0.92 | | 1.72 (0.32–9.32) 0.53, 0.73 | | 0.55 (0.14–2.16) 0.39, 0.63 | | 0.47 (0.12–1.85) 0.28, 0.50 |  |  |
| **Household size** | |  | |  | |  | |  |  |  |
| 4 and below | | Ref | | Ref | | Ref | | Ref |  |  |
| Above 4 | | 1.13 (0.59–2.16) 0.71, 0.85 | | 1.51 (0.87–2.62) 0.14, 0.32 | | 1.05 (0.52–2.12) 0.90, 0.95 | | 0.85 (0.39–1.86) 0.69, 0.83 |  |  |
| **Polygamous marriage with deceased husband** | | | |  | |  | |  |  |  |
| No | | Ref | | Ref | | Ref | | Ref |  |  |
| Yes | | 1.25 (0.67–2.32) 0.48, 0.70 | | 1.08 (0.63–1.84) 0.79, 0.89 | | 1.24 (0.64–2.42) 0.53, 0.73 | | 1.58 (0.76–3.28) 0.22, 0.41 |  |  |
| **Christian denominations** | |  | |  | |  | |  |  |  |
| Catholic | | Ref | | Ref | | Ref | | Ref |  |  |
| African traditional churches | | 1.27 (0.51–3.17) 0.61, 0.77 | | 0.84 (0.37–1.87) 0.66, 0.82 | | 0.67 (0.26–1.73) 0.41, 0.64 | | 1.09 (0.40–2.94) 0.86, 0.93 |  |  |
| Non-denominational churches | | 1.16 (0.47–2.90) 0.74, 0.87 | | 1.02 (0.47–2.21) 0.96, 0.97 | | 0.88 (0.36–2.15) 0.77, 0.89 | | 0.74 (0.26–2.10) 0.58, 0.76 |  |  |
| Protestant | | 0.42 (0.13–1.30) 0.13, 0.31 | | 0.51 (0.21–1.24) 0.14, 0.32 | | 0.36 (0.12–1.10) 0.07, 0.23 | | 0.49 (0.15–1.58) 0.23, 0.44 |  |  |
| **Current economic activity** | |  | |  | |  | |  |  |  |
| Farming | | Ref | | Ref | | Ref | | Ref |  |  |
| Casual labor | | **3.48 (1.33–9.07) 0.01, 0.04** | | 2.15 (0.95–4.88) 0.07, 0.16 | | 1.98 (0.76–5.13) 0.16, 0.33 | | 2.72 (1.01–7.30) 0.05, 0.13 |  |  |
| Employed | | 1.54 (0.08–29.30) 0.77, 0.97 | | 0.71 (0.04–13.18) 0.82, 0.92 | | 1.04 (0.06–19.46) 0.98, 0.98 | | 1.43 (0.08–27.07) 0.81, 0.92 |  |  |
| Microbusiness | | **3.24 (1.59–6.60) <0.01, <0.01** | | 1.49 (0.82–2.72) 0.19, 0.36 | | 0.93 (0.42–2.02) 0.85, 0.94 | | 1.14 (0.49–2.66) 0.76, 0.96 |  |  |
| Supported by others†† | | 0.84 (0.21–3.34) 0.80, 0.97 | | 0.39 (0.10–1.46) 0.16, 0.32 | | 0.56 (0.15–2.18) 0.41, 0.66 | | 1.11 (0.33–3.71) 0.87, 0.95 |  |  |
| **Home ownership** | |  | |  | |  | |  |  |  |
| Yourself | | Ref | | Ref | | Ref | | Ref |  |  |
| Deceased spouse | | 0.48 (0.21–1.09) 0.08, 0.24 | | 0.62 (0.30–1.29) 0.20, 0.40 | | 0.36 (0.16–0.81) 0.01, 0.07 | | 0.38 (0.17–0.85) 0.02, 0.09 |  |  |
| Marital relatives† | | 1.35 (0.57–3.19) 0.49, 0.70 | | 1.33 (0.60–2.95) 0.49, 0.70 | | 0.79 (0.32–1.94) 0.61, 0.77 | | 0.36 (0.12–1.05) 0.06, 0.20 |  |  |
| **Agricultural land ownership** | |  | |  | |  | |  |  |  |
| Yourself | | Ref | | Ref | | Ref | | Ref |  |  |
| Deceased spouse | | 0.51 (0.21–1.25) 0.14, 0.32 | | 0.97 (0.39–2.39) 0.95, 0.97 | | 0.43 (0.17–1.07) 0.07, 0.22 | | 0.51 (0.20–1.32) 0.16, 0.35 |  |  |
| Marital relatives† | | 0.67 (0.27–1.68) 0.40, 0.63 | | 1.11 (0.44–2.81) 0.83, 0.92 | | 0.51 (0.19–1.31) 0.16, 0.35 | | 0.41 (0.14–1.17) 0.10, 0.27 |  |  |
| **Deceased husband’s HIV status** | | | |  | |  | |  |  |  |
| Negative | | Ref | | Ref | | Ref | | Ref |  |  |
| Positive | | 2.01 (0.91–4.42) 0.08, 0.25 | | 1.45 (0.75–2.80) 0.27, 0.49 | | 1.27 (0.55–2.97) 0.57, 0.76 | | 1.74 (0.75–4.02) 0.20, 0.40 |  |  |
| Don't Know | | **4.10 (1.99–8.44) <0.01, <0.01** | | 1.81 (0.93–3.52) 0.08, 0.24 | | 2.20 (1.01–4.79) 0.05, 0.17 | | 1.89 (0.79–4.52) 0.15, 0.34 |  |  |
| **Widows’ HIV status** |  | |  | |  | |  | | |  |
| Negative | Ref | | Ref | | Ref | | Ref | | |  |
| Positive | 1.62 (0.88–3.01) 0.12, 0.15 | | 1.25 (0.73–2.16) 0.42, 0.47 | | 1.68 (0.86–3.26) 0.13, 0.15 | | 2.01 (0.99–4.09) 0.05, 0.07 | | |  |
| AOR = Adjusted Odds Ratio, CI = Confidence Interval; p = raw p-values that are unadjusted for multiple testing; q = false discovery rate adjusted p-values were calculated using Benjamini-Hochberg procedure ; †Marital relatives: children, other family members or persons in the community; ††Includes those who reported no income activity or reported relying on support from family or government for means of livelihood | | | | | | | | |  |  |
